# Treatment outcomes in a safety observational study of dihydroartemisinin/piperaquine (Eurartesim^®^) in the treatment of uncomplicated malaria at public health facilities in four African countries

**DOI:** 10.1186/s12936-016-1099-7

**Published:** 2016-01-27

**Authors:** Alexander Adjei, Solomon Narh-Bana, Alberta Amu, Vida Kukula, Richard Afedi Nagai, Seth Owusu-Agyei, Abraham Oduro, Eusebio Macete, Salim Abdulla, Tinto Halidou, Ali Sie, Isaac Osei, Esperance Sevene, Kwaku-Poku Asante, Abdunoor Mulokozi, Guillaume Compaore, Innocent Valea, Martin Adjuik, Rita Baiden, Bernhards Ogutu, Fred Binka, Margaret Gyapong

**Affiliations:** Dodowa Health Research Centre, Dodowa, Ghana; Kintampo Health Research Centre, Kintampo, Ghana; Navrongo Health Research Centre, Navrongo, Ghana; Centro de Investigação em Saúde de Manhiça (CISM), Manhiça, Mozambique; Ifakara Health Institute, Ifakara, Tanzania; Nanoro Health Research Centre, Nanoro, Burkina Faso; Nouna Health Research Centre, Nouna, Burkina Faso; INDEPTH-Network, Accra, Ghana; University of Science and Allied Sciences, Ho, Ghana

**Keywords:** Dihydroartemisinin-piperaquine, Uncomplicated malaria, Artemisinin-based combination therapy, Treatment outcome, Effectiveness

## Abstract

**Background:**

Dihydroartemisinin-piperaquine (DHA-PQ) is one of five WHO recommended artemisinin combination therapy (ACT) for the treatment of uncomplicated malaria. However, little was known on its post-registration safety and effectiveness in sub-Saharan Africa. DHA-PQ provides a long post-treatment prophylactic effect against re-infection; however, new infections have been reported within a few weeks of treatment, especially in children. This paper reports the clinical outcomes following administration of DHQ-PQ in real-life conditions in public health facilities in Burkina Faso, Ghana, Mozambique, and Tanzania for the treatment of confirmed uncomplicated malaria.

**Methods:**

An observational, non-comparative, longitudinal study was conducted on 10,591 patients with confirmed uncomplicated malaria visiting public health facilities within seven health and demographic surveillance system sites in four African countries (Ghana, Tanzania, Burkina Faso, Mozambique) between September 2013 and April 2014. Patients were treated with DHA-PQ based on body weight and followed up for 28 days to assess the clinical outcome. A nested cohort of 1002 was intensely followed up. Clinical outcome was assessed using the proportion of patients who reported signs and symptoms of malaria after completing 3 days of treatment.

**Results:**

A total of 11,097 patients were screened with 11,017 enrolled, 94 were lost to follow-up, 332 withdrew and 10,591 (96.1 %) patients aged 6 months–85 years met protocol requirements for analysis. Females were 52.8 and 48.5 % were <5 years of age. Malaria was diagnosed by microscopy and rapid diagnostic test in 69.8 % and 29.9 %, respectively. At day 28, the unadjusted risk of recurrent symptomatic parasitaemia was 0.5 % (51/10,591). Most of the recurrent symptomatic malaria patients (76 %) were children <5 years. The mean haemoglobin level decreased from 10.6 g/dl on day 1 to 10.2 g/dl on day 7. There was no significant renal impairment in the nested cohort during the first 7 days of follow-up with minimal non-clinically significant changes noted in the liver enzymes.

**Conclusion:**

DHA-PQ was effective and well tolerated in the treatment of uncomplicated malaria and provides an excellent alternative first-line ACT in sub-Saharan Africa.

## Background

Artemisinin-based combination therapy (ACT) is highly effective for the treatment of uncomplicated falciparum malaria with five formulations, including dihydroartemisinin (DHA) and piperaquine (PQ) combination currently recommended by the World Health Organization (WHO) [1]. ACT has been adopted as first-line treatment in 79 of 88 countries where *Plasmodium falciparum* is endemic as at 2013 [2].

The different modes of action of the partner drugs contribute to the efficacy of ACT. These partner drugs (e.g., PQ, mefloquine, amodiaquine, lumefantrine, pyronaridine) are eliminated slowly compared to the artemisinin derivatives (e.g., DHA, artesunate, artemether), which are eliminated rapidly, although they swiftly bring down the parasite biomass [3]. This provides protection for the artemisinin derivative while the partner drugs provide prolonged drug cover [4].

The half-life of PQ is 21–28 days [5–7], longer than most of the partner drugs used in ACT, providing a longer post-treatment prophylactic effect compared to lumefantrine (3–5 days) [3, 8], the active amodiaquine metabolite desethylamodiaquine (7–12 days) [9], mefloquine (17–24 days) [10], and pyronaridine (13.2 and 9.6 days in adults and children, respectively) [11]. The long half-life of PQ has an important role in preventing re-infection or recrudescence. It may also contribute to recovery of decreased haemoglobin (Hb), which has been observed in the pathogenesis of malaria especially on days 3 and 7, although in a majority of patients Hb recovery to normal levels occurs on day 28 [12]. Pooled analysis of seven randomized, controlled trials on ACT, including Dihydroartemisinin-piperaquine (DHA-PQ) from 14 sub-Saharan African sites of 3044 children ≤5 years showed a decline in Hb levels on day 7 with recovery between days 14–28 [13].

Despite the longer half-life of PQ, new infections have been reported within a few weeks of treatment with DHA-PQ, particularly in children age 2–10 years because of their higher body weight-normalized clearance. This leads to lower PQ exposure compared to older persons with lower day 7 PQ concentrations after standard weight-based dosing [14, 15]. This effect has been associated with an increased risk of recurrent parasitaemia [14].

Pooled analysis of 14 studies published in 13 articles (11 in Southeast Asia, one in China and one in Rwanda) involving 2636 patients on DHA-PQ from 2002 to 2006 found it to be safe and highly effective in the treatment of uncomplicated falciparum malaria [16] and comparable to other ACT [14, 17, 18]. Treatment with DHA-PQ in Africa has shown a lower risk of recurrent malaria compared to artesunate and amodiaquine combination (AS/AQ) [19] and artemether and lumefantrine combination (AL) [18, 20] but some studies showed no significant prophylactic effect in comparison to AL [21].

This study evaluated the clinical outcomes of DHQ-PQ when administered under real-life conditions for the treatment of confirmed uncomplicated malaria in public health facilities in Burkina Faso, Ghana, Mozambique, and Tanzania as part of a larger study conducted to assess the clinical safety of DHA-PQ (Eurartesim^®^) in Africa [22].

## Methods

### Study design

This was an observational, non-comparative, longitudinal study in patients with signs and symptoms of uncomplicated malaria confirmed by a parasitological diagnosis or, when the tests were not available, by WHO diagnostic criteria in seven health and demographic surveillance system (HDSS) sites in four sub-Saharan African countries from September 2013 to April 2014. The detailed study design together with the safety profile has been published elsewhere [22].

### Study sites

The seven study sites are part of eight HDSS INDEPTH Effectiveness and Safety (INESS) platforms established since 2009 with Bill and Melinda Gates Foundation funding. The sites are Dodowa, Kintampo and Navrongo in Ghana, Nanoro and Nouna in Burkina Faso, Manhiça in Mozambique, and Rufiji in Tanzania. The other member site is Ifakara in Tanzania. They are all members of WHO Programme for International Drug Monitoring with spontaneous and cohort event monitoring on anti-malarials, alongside their routine, longitudinal, health and demographic surveillance data gathering [22]. Estimated total population of these seven HDSS sites is 752,937 [23]. Malaria is endemic in all the study sites with varying transmission due to different geographical conditions and rainfall patterns. *P. falciparum* is responsible for the majority of malaria cases at these sites. *Anopheles gambiae* and *A. funestus* are the main vectors. AS/AQ and AL are first-line ACT for the treatment of uncomplicated malaria, with only Ghana having DHA-PQ as the third first-line. Detailed profiles of the study sites are published elsewhere [24–30].

### Recruitment process

Patients with confirmed uncomplicated malaria were recruited from the outpatient departments of selected public health facilities in the seven HDSS sites. The inclusion criteria were age ≥6 months, weight ≥5 kg, ability to tolerate oral medications, and willingness to participate based on signed informed consent. Malaria was diagnosed as per the national policies in the four countries in line with WHO recommendations [history of fever in the previous 24 h or presence of fever and parasitological confirmation by microscopy or rapid diagnostic test (RDT)]. The exclusion criteria included: known allergy to artemisinin or to PQ; history of taking a DHA-PQ dose in the previous 4 weeks, known pregnancy, lactating women, complicated malaria, history of taking medicinal products that are known to prolong the corrected QT (QTc) interval on echocardiogram (ECG) (including anti-arrhythmics, neuroleptics and certain antimicrobial agents), and family history of sudden unexplained death or personal/family history of predisposing cardiac conditions for arrhythmia/QT prolongation. Patients or their guardians were encouraged to comply with all scheduled follow-up visits.

### Main group and nested cohort

Patients were recruited into two groups, the main and nested cohort with no special criteria apart from the stated inclusion and exclusion criteria. Sites determined the cohort criteria based on their follow-up plan. Each eligible patient had a detailed clinical assessment including past medical and drug history after a confirmed parasitological (microscopy and RDT) diagnosis. Capillary blood sample was used in preparing thick blood smear and parasite density calculated per 200 leucocytes. The sites used different HRP2-based RDTs with sensitivity ranging from 90.5–99.7 % for *P. falciparum*, 92.9–95.5 % for non-*P. falciparum* and specificity of 97.5–99.5 %.

### Nested cohort laboratory procedure

A detailed laboratory assessment beside a prerequisite confirmed microscopic parasitaemia at baseline was carried out for the nested cohort. Investigations carried out were haematology (white cell count with differentials, red blood cells, Hb level and platelet count), biochemistry [total bilirubin, alanine aminotransferase (ALT), aspartate aminotransferase (AST), creatinine, urea, serum potassium and chloride], and plasma concentration of PQ as well as triplicate baseline ECG before day 1 drug administration. On each visit, 5 ml of venous blood was used for the investigations. Different types of Sysmex (Poch 100i, KX-2IN, XT 20090i) and ABX micros B were used for haematology at the various sites while Selectra Junior, Humalyzer primus with Integra 400 plus and Vitalab flexor E were used for biochemistry analysis of patient samples.

### Drug administration

Eurartesim^®^ (DHA-PQ) was administered once daily based on body weight for 3 days. Two concentrations were used: 20/160 and 40/320 mg of DHA and PQ, respectively, as shown in Table 1. Drugs were administered under direct observed therapy (DOT) on the first day for all patients and for the 3 days in the nested cohort. Drug was administrated with water and patients observed for an hour in case of vomiting and any other adverse events (AEs). Those who vomited within 30 min were re-administered the full dose and those who vomited after 30 min but within the hour were given half of the dose. Re-dosing was done once and rescue treatment given for unsuccessful re-dosing as per the specific national guidelines. Patients were encouraged to complete the treatment, avoid high fatty or calorie diets 3 h before and after dosing. Paediatric doses were however crushed and administered with water.

**Table 1:** Drug concentrations

Body weight (kg)	Daily dosage (mg)	Number of tablets per dose	
		20/160 mg DHA-PQ	40/320 mg DHA-PQ
5 to <7	10 mg DHA and 80 mg PQ	^1^/_2_ tablet	
7 to <13	20 mg DHA and 160 mg PQ	1 tablet	
13 to <24	40 mg DHA and 320 mg PQ		1 tablet
24 to <36	80 mg DHA and 640 mg PQ		2 tablets
36 to <75	120 mg DHA and 960 mg PQ		3 tablets
75 to <100	160 mg DHA and 1280 mg PQ		4 tablets

### Follow-up procedure

Patients were followed up for 28 days with active follow-up on day 5 (±2 days) and day 28 by telephone or physical contact to document recovery status and AEs. Compliance to treatment was assessed in the main group by asking if patient adhered to the study prescription on day 5 (±2 days). The nested cohort was additionally followed on days 2, 3 and 7. Day 2 visit was at home, for DOT of the second dose of the study medication, monitor clinical status and record any AEs. Patients were seen at the health facilities on day 3 for treatment completion, ECG recording (single pre and 3- to 4-hour triplicate post dosage) and then repeat haematology, biochemistry and plasma PQ concentration. These investigations were repeated on day 7 with a single ECG recording. Each facility visit included detailed clinical workup, screening for AEs and appropriate case report forms were completed. Patients were also encouraged to report any AEs experienced from day 1 to day 28 by telephone or visit to the health facility (unscheduled visit). Patients with signs and symptoms of malaria on all unscheduled visits were re-tested for malaria and managed appropriately as per national guidelines.

### Outcome

The primary outcome of interest was adequate clinical response at day 28, defined as absence of clinical signs and symptoms for malaria at day 28. Treatment failure was defined as unresolved symptoms and signs and parasitaemia within 2 weeks of full treatment or recurrence of fever and parasitaemia after 2 weeks of full treatment [1]. The definition was used to estimate the rate of unadjusted recurrent symptomatic parasitaemia. Secondary outcomes included the prevalence of fever, change in mean Hb levels, mean bilirubin levels, liver enzymes, and renal function on the nested cohort during the first 7 days of follow-up.

### Data entry and statistical analysis

Data were double-entered using Open Clinica software from all sites. Statistical analyses were performed using the software package STATA^®^ (version 11.2). Descriptive analysis of all data recorded at study entry was carried out to characterize the population studied. The main outcome of interest, frequencies and respective percentages were calculated. Proportions, means and standard deviations were used in estimating the secondary outcomes. Means were estimated for other continuous independent variables. The parasite densities were grouped as <500 parasites**/**µL, 500 to <5000 parasites**/**µL and 5000 parasites**/**µL and above.

### Ethics statement

The Institutional Ethics Committee of all seven HDSS sites involved in the trial and the Independent National Ethics Review Committees in these four countries approved the protocol. The protocol and case report forms are available and annexed as supporting documents. The study was conducted in accordance with Good Clinical Practice and the Helsinki declaration for biomedical research on human subjects. The Safety Committee, Central Data Monitoring Team and Independent Monitors provided oversight of study conduct. The study was registered with Clinicaltrials.gov; Trial registration number NCT02199951.

## Results

**Fig. 1:**
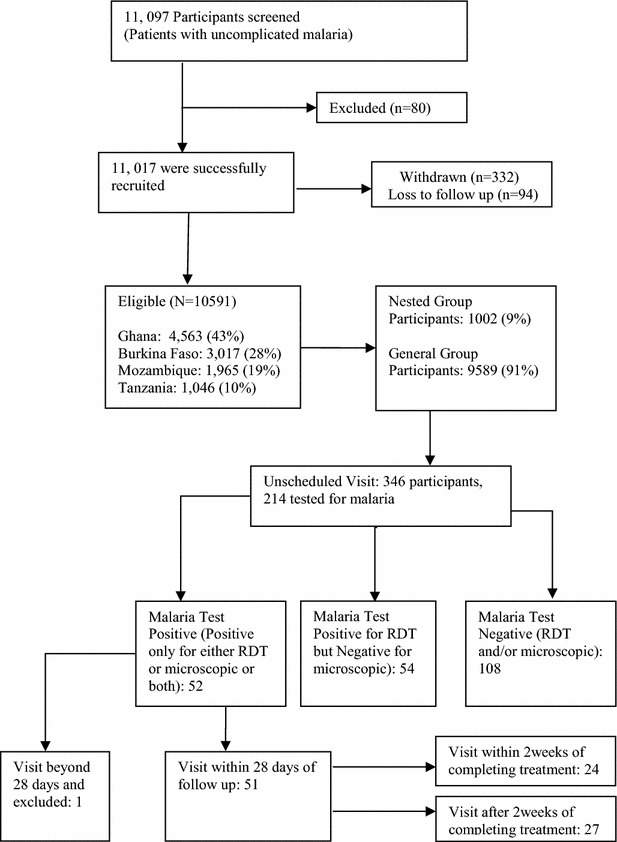
Study flow chart

A total of 10,591 participants successfully completed the study and were included in the analysis (Fig. 1). The age distribution was 6 months–85 years with a median of 8 years. About 53 % (5068) of the participants were females and 48.4 % (4654) children <5 years of age. Patients were recruited at three HDSS sites in Ghana, two in Burkina Faso and one each in Tanzania and Mozambique. Manhiça site had the highest recruitment of 18.6 % (1965) with Dodowa site recruiting the least, 8.4 % (884). The highest recruitment in the nested cohort and the main group were 27.4 % (274) and 19.6 % (1876) from Navrongo and Manhiça sites, respectively (Table 2).

**Table 2:** The demographic characteristics of the recruited participants from the seven HDSS sites

Variable	Main group n (%)	Nested cohort n (%)	Total n (%)
Age			
6–11 months	327 (3.4)	6 (0.6)	333 (3.1)
12–59 months	4327 (45.1)	325 (32.4)	4652 (43.9)
Above 59 months	4935 (51.5)	671 (67.0)	5606 (52.9)
Enrolment			
Eligible	9589 (98.0)	1002 (76.2)	10,591 (95.4)
Excluded	68 (0.7)	12 (0.9)	80 (0.7)
Withdrawn	125 (1.3)	301 (22.9)	426 (3.8)
Sex			
Male	4521 (47.2)	489 (48.2)	5004 (47.3)
Female	5068 (52.8)	518 (51.7)	5586 (52.7)
Country/site			
Burkina faso			3017 (28.5)
Nouna	1658 (17.3)	102 (10.2)	1760 (16.6)
Nanoro	1060 (11.1)	197 (19.7)	1257 (11.9)
Ghana			4563 (43.1)
Dodowa	729 (7.6)	155 (15.6)	884 (8.4)
Kintampo	1763 (18.4)	15 (1.5)	1778 (16.8)
Navrongo	1627 (17.0)	274 (27.4)	1901 (17.9)
Mozambique			1965 (18.6)
Manhiça	1876 (19.6)	89 (8.9)	1965 (18.6)
Tanzania			1046 (9.9)
Rufiji	876 (9.1)	170 (17.0)	1046 (9.9)

The mean weight and height for the main group were 26.0 kg and 117.2 cm, and that for the nested cohort were 28.7 kg and 125.5 cm, respectively. Weight and height measurements were taken on day 1. The mean temperature for both cohorts on day 1 was 37.4 °C and declined to 36.6 °C on day 7 for the nested cohort. Out of the 1002 patients in the nested cohort, 441 had temperature ≥37.5 °C on day 1. This decreased to 35 and 39 patients on day 3 and day 7, respectively. Forty-three per cent (4104) of the main group had fever on day 1. With the exception of blood pressure, all other vital signs decreased with age and no significant change was recorded among the nested cohort within the first 7 days of follow-up.

### Laboratory results

Malaria diagnosis was confirmed by microscopy and RDT in 69.8 % (7391) and 29.9 % (3170) patients, respectively, with 30 patients diagnosed clinically. The RDTs were *P. falciparum* cases. There were 94 cases of mixed infections from the microscopic findings: 90 of *P. falciparum* and *P. malariae* and four of *P. falciparum* and *P. ovale* with no case of *P. vivax* reported. Gametocyte carriage was 2.7 % (199/7260) and 11.1 % (13/117) for *P. falciparum* and *P. malariae* cases, respectively, by microscopy. Majority of patients had parasitaemia <5000/µL (Table 3).

**Table 3:** Malaria parasites species determined by microscopy

Parasites density (**/**µL)	*Plasmodium* species n (%)					
	*P. falciparum*		*P. ovale*		*P. malariae*	
	Total	Nested	Total	Nested	Total	Nested
	7260 (98.2)	995	14 (0.2)	3	117 (1.6)	25
<500	1949 (26.8)	302	3 (21.4)	0	52 (44.4)	11
500 to <5000	2028 (27.9)	265	10 (71.4)	3	60 (51.3)	13
≥5000	3283 (45.2)	428	1 (7.1)	0	5 (4.3)	1

The nested cohort total is more than the 1002 because of mixed infections

### Haematology results for nested cohort (n = 1002)

**Fig. 2:**
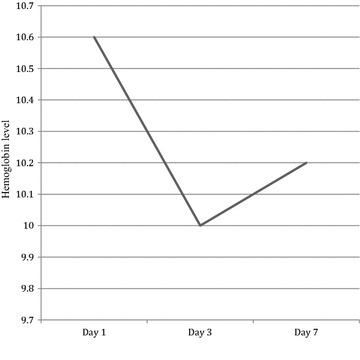
Mean haemoglobin level (g/dl) in the nested cohort (n = 1002)

The mean Hb of the nested cohort decreased from 10.6 g/dl on day 1 to 10.2 g/dl on day 7, however, it increased by 0.2 g/dl from day 3 to day 7 (Fig. 2). A total of 33 % (326) of patients had anaemia (Hb <10 g/dl) on day 1 with one having severe anaemia (Hb 4.6 g/dl). Even though this patient should not have been enrolled as per the protocol, the Hb remained between 4.5 and 4.6 g/dl from enrolment to day 7. The number of patients with anaemia increased to 47 % (471) and 43 % (431) on days 3 and 7, respectively. Seventy-five per cent or more of patients had Hb of ≥8 g/dl on days 1 (77 %), 3 (75 %) and 7 (77 %). A total of 17 % (174) and 16 % (159) patients had a decline in Hb to levels ≤10 g/dl on days 3 and 7, respectively, despite being ≥10 g/dl on day 1.

### Clinical chemistry results for nested cohort (n = 1002)

There was no significant liver or renal impairment in the nested cohort with the mean values decreasing from day 1 to day 7 (Table 4). A total of 345 patients who were enrolled had bilirubin levels >18 μmol/L on day 1 with one patient having 174.9 μmol/L. Out of these, 87 % (299/345) had their bilirubin levels decreased below the upper limit (18 μmol/L) on day 7. In all, 76 patients on day 7 still had elevated bilirubin levels with maximum value recorded being 71.3 μmol/L and 28 % (21/76) had levels twice the upper limit. ALT and AST mean levels were all within normal (<40 U/L), however, 150 and 164 patients had their baseline ALT and AST values higher than 40 U/L, respectively, on day 1. Of these patients, 67 % (101/150) and 64 % (105/164) had their ALT and AST levels return to normal on day 7, respectively. However, there were 68 and 49 patients who had normal ALT and AST levels (<40 U/L), respectively, on day 1 but higher than normal on day 7. The total number of patients with elevated ALT and AST levels on day 7 were 115 and 106 patients, respectively. Notably, 17 % (19/115) and 12 % (13/106) had levels higher than twice the upper limit of 40 U/L on day 7. Measured mean blood urea levels decreased from day 1 to day 7 while there was a marginal increase of 1.1 in mean creatinine level from day 1 to day 3 and then a decrease by 2.2 on day 7 (Table 4).

**Table 4:** Biochemistry results for the nested cohort (n = 1002)

Variable (unit)	Day 1		Day 3		Day 7	
	Mean (SD)	n (%)	Mean (SD)	n (%)	Mean (SD)	n (%)
Bilirubin (μmol/L)	17.8 (15.0)	995 (99.3)	8.8 (8.0)	994 (99.2)	9.2 (8.1)	1000 (99.8)
ALT (U/L)	28.9 (28.8)	997 (99.5)	27.3 (28.5)	995 (99.3)	27.7 (24.4)	1000 (99.8)
AST (U/L)	31.1 (36.4)	1000 (99.8)	29.0 (30.3)	997 (99.5)	27.9 (21.2)	1001 (99.9)
Creatinine (μmol/L)	52.0 (30.2)	990 (98.8)	53.1 (32.5)	984 (98.2)	50.9 (29.4)	994 (99.2)
Urea (mmol/L)	4.9 (3.9)	997 (99.5)	4.3 (3.9)	983 (98.1)	4.1 (4.1)	998 (99.6)

### Unscheduled visits by patients with clinical signs and symptoms

**Fig. 3:**
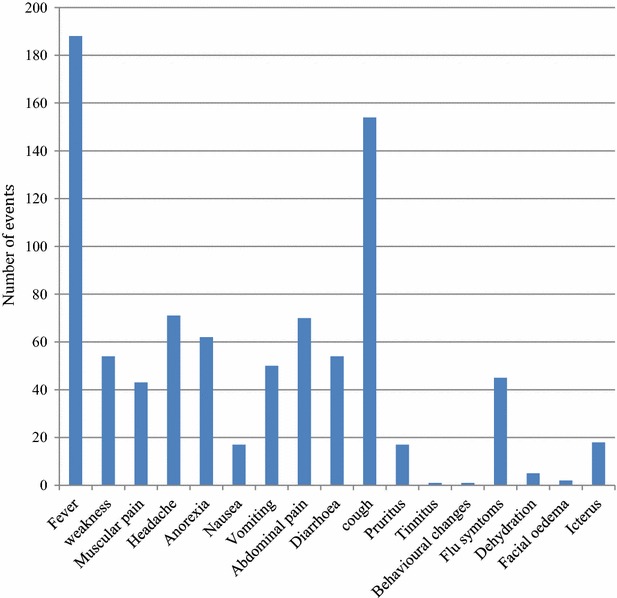
Symptoms presented by participants on unscheduled visits

A total of 346 patients came for unscheduled visits of which 16 had a repeat visit (unscheduled visit two) from day 4 to day 28. Fever, cough, headache, abdominal pains, and anorexia were the common symptoms presented on these visits (Fig. 3). Of the unscheduled visits 65 % (214/330) had malaria test done by microscopy, RDT or both methods. Of those tested, 24 % (51/214) were positive for malaria (24 RDT or 19 microscopy or eight from both methods) and 25 % (54/214) as false positive (RDT positive but microscopic negative). Majority of the patients who tested positive (48/51) were in the main group with only two patients yet to complete day-3 dosage when contacted on day 5 (±2 days) follow-up. They were treated with AS/AQ (14/51), AL (19/51), quinine (7/51), and no anti-malarial treatment (11/51). Of the patients who received no treatment, 73 % (8/11) sought care within 2 weeks from day of enrolment and even though still tested positive by RDT, they were treated for acute respiratory infections (ARI) and gastroenteritis based on other symptoms and signs presented.

### Primary outcome

**Fig. 4:**
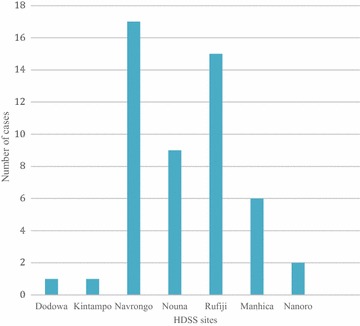
Distribution of recurrent symptomatic parasitaemia cases among the seven sites

By day 28 the unadjusted risk of recurrent symptomatic parasitaemia was 0.5 % (51/10,591). Of these, 47 % (24/51) were seen within 2 weeks of treatment (4–14 days) (median 7.7 days) and 53 % (27/51) after 2 weeks of treatment (15–28 days) (median 21 days). Most patients 76 % (39/51) with recurrent symptomatic parasitaemia were <5 years. Navrongo, Nouna and Rufiji sites recorded 80 % (33, 18 and 29 %, respectively) of these cases as shown in (Fig. 4). Using univariate analysis, temperature at first presentation, age and some of the study sites were significantly associated with treatment failure. A 1 °C increase in temperature was 13 % more likely to lead to treatment failure (OR I.13, CI 95 %, 0.88–1.45). Patients aged >5 years were 27 % (OR 0.27, CI 95 %, 0.14–0.52) less likely to develop treatment failure as compared to <5 years. Patients recruited from Navrongo, Rufiji and Nouna sites were eight, 13 and five times more likely to have treatment failure than those recruited from Dodowa site, respectively (OR 7.97, CI 95 %, 1.06–59.96; OR 12.85, CI 95 %, 1.69–97.45; OR 4.54, CI 95 %, 0.57–35.88). Being in the main group was 60 % (OR 0.60, CI 95 %, 0.16–1.92) more likely to develop treatment failure compared to those in the nested cohort. After adjusting for demographic factors, clinical presentation and baseline parasitaemia: age (>5 years) and one study site (Rufiji) were significantly associated with treatment failure (OR 0.30, CI 95 %, 0.15–0.58; OR 8.49, CI 95 %, 1.10–65.47).

## Discussion

In this large-scale, phase IV study of DHA-PQ (Eurartesim) administration in ‘real-life clinical situations’ at public health facilities for the treatment of confirmed uncomplicated malaria, WHO acceptable (>95 %) adequate clinical response with very low unadjusted rate of recurrent symptomatic parasitaemia of 0.5 % by day 28 was observed. This rate is lower than previously observed in more controlled studies in Southeast Asia [16] and sub-Saharan Africa [18, 19, 31] but may be due to the fact that non-symptomatic parasitaemia was not sought. However, this is a reflection of routine clinical care and emphasizes the fact that true treatment outcomes are not best measured under safety studies but rather through well-designed efficacy clinical trials.

This study found that the majority of the recurrent symptomatic parasitaemia occurred almost in equal proportions within 14 days and after 14 days’ post-treatment. This is in contrast to the review of 39 trials on ACT in the WHO 2010 treatment guidelines, which had enrolled 6124 participants. No treatment failure was recorded within two weeks in 32 of the trials. In the remaining seven trials, failure rates at day 14 ranged from 1–7 %, highlighting unusual rate of treatment failure within 2 weeks of treatment [1]. This could be due to PCR-corrected cure rates and also the well-documented, longer, post-treatment prophylactic effect of DHA-PQ because of the longer half-life of PQ [5–7] and relatively short follow-up of 28 days in this study. The high treatment failure rates recorded in Navrongo and Rufiji could be due to intense malaria transmission during the study period compared to other sites, particularly Dodowa. The intensive follow-up in the nested cohort (DOT on the 3 days) clearly showed in the number of patients (3/51) who had treatment failure and this emphasizes the importance of drug adherence.

The majority of treatment failure cases were found in the <5 age group corroborating earlier reports [14]. In younger patients it has been documented that they may have a lower exposure to PQ drug level at current dosing, predisposing them to recurrent parasitaemia in DHA-PQ studies [14, 15]. Documented evidence from the Worldwide Antimalarial Resistance Network (WWARN) DHA-PQ Study Group showed that increasing the minimal dosage of PQ in <5 years reduces the risk of treatment failure [32]. This has led to the revised dose recommendation by WHO in the 2015 treatment guideline for malaria in children with weight <25 kg [33]. The recurrent parasitaemia in the younger age group could also be due to the lack of immunity due to minimal prior exposure to malaria. This study reveals DHA-PQ to be very well tolerated with excellent adherence because of the simple daily dosing, as previously described [16, 31]. Other studies in some parts of Africa have documented the excellent efficacy of DHA-PQ with good tolerability, prolonged prophylactic effect and once daily dosage, making it an excellent ACT for high transmission zones [14].

Of the 51 patients who tested positive during the unscheduled visit, 22 % (11/51) were not treated with an anti-malarial contrary to the WHO malaria treatment guideline [1]. The reason for the non-treatment of these patients, the majority (73 %) of whom were within 2 weeks of treatment and still tested positive by RDT, was because of the long clearance time of the RDTs used. They were treated for other presenting conditions (ARI and gastroenteritis). The HRP2-based RDT is known to remain positive within 2 weeks of treatment, or sometimes more, after effective treatment because of the persistence of malaria antigens [34] leading to false positive test. A limitation of this study was not microscopically confirming all recurrent symptomatic cases because of the possibility of false positive from RDT only, but this is what happens in real-life situations where different WHO-recommended diagnostic methods are used.

The mean Hb outcome on day 7 was lower than the baseline level. This confirms similar results by Tjitra et al. [12] and Olliaro et al. [13], and can be attributed to disease pathogenesis with loss of infected and malaria antigen-coated red blood cells during treatment and recovery. The long half-life of PQ, which is known to prevent relapse or re-infection, has also been suggested to contribute to the recovery from anaemia together with many other factors [12]. Some studies have reported increase in Hb levels from day 7 to 28 after ACT [35]. The mean values of the clinical chemistry parameters were within normal reference ranges and further decreased during the first seven days of follow-up, which is comparable to other studies [13]. There were patients who were observed to have recorded values higher on day 7 than the baseline and vice versa. It will be difficult to attribute these observed changes only to the drug, knowing the well-documented effect of malaria parasites on the liver and kidney [36, 37] and possibility of co-infections, especially viral.

## Conclusion

This is the largest phase IV study in sub-Saharan Africa on DHA-PQ for the treatment of uncomplicated malaria, showing that DHA-PQ is very effective and tolerable. DHA-PQ should be an appropriate alternative to other first-line ACT in sub-Saharan Africa and its deployment will reduce drug pressure on the two widely used ACT (AS/AQ and AL).

## Abbreviations

ACT: Artemisinin-based combination therapy
DHA: Dihydroartemisinin
PQ: Piperaquine
WHO: World Health Organization
Hb: Haemoglobin
AS/AQ: Artesunate and amodiaquine combination
AL: Artemether and lumefantrine combination
HDSS: Health and Demographic surveillance system
INESS: INDEPTH Effectiveness and Safety
RDT: Rapid diagnostic test
ALT: Alanine aminotransferase
AST: Aspartate aminotransferase
ECG: Echocardiogram
DOT: Direct observed therapy
AEs: Adverse events
ARI: Acute respiratory infections
WWARN: Worldwide Antimalarial Resistance Network
